# Effect of Structurally Different Pectin on Dough Rheology, Structure, Pasting and Water Distribution Properties of Partially Meat-Based Sugar Snap Cookies

**DOI:** 10.3390/foods10112692

**Published:** 2021-11-04

**Authors:** Asad Nawaz, Enpeng Li, Ibrahim Khalifa, Noman Walayat, Jianhua Liu, Nilofar, Hafiz Muhammad Ahsan, Sana Irshad, Hassan Barakat, José M. Lorenzo, Mirian Pateiro, Shahida Anusha Siddiqui, Muhammad Inam-Ur-Raheem

**Affiliations:** 1 Key Laboratory of Plant Functional Genomics of the Ministry of Education/Jiangsu Key Laboratory of Crop Genomics and Molecular Breeding, College of Agriculture, Yangzhou University, Yangzhou 225009, China; 007298@yzu.edu.cn; 2 Co-Innovation Center for Modern Production Technology of Grain Crops, Yangzhou University, Yangzhou 225009, China; 3 Food Technology Department, Faculty of Agriculture, Benha University, Moshtohor 13736, Egypt; Ibrahiem.khalifa@fagr.bu.edu.eg (I.K.); Haa.mohamed@qu.edu.sa (H.B.); 4 College of Food Science and Technology, Zhejiang University of Technology, Hangzhou 310014, China; Jhliu@zjut.edu.cn; 5 Department of Biological Science and Technology, College of Life and Materials Systems Engineering, Tokushima University, Tokushima 770-0000, Japan; nilofar.tokushima@yahoo.com; 6 Institute of Food Science and Nutrition, Bahauddin Zakariya University, Multan 60000, Pakistan; ahsansmeer@gmail.com; 7 School of Environmental Studies, China University of Geo Sciences, Wuhan 430074, China; sanairshad55@gmail.com; 8 Department of Food Science and Human Nutrition, College of Agriculture and Veterinary Medicine, Qassim University, Buraydah 51452, Saudi Arabia; 9 Centro Tecnológico de la Carne de Galicia, 32900 Ourense, Spain; jmlorenzo@ceteca.net (J.M.L.); mirianpateiro@ceteca.net (M.P.); 10 Área de Tecnología de los Alimentos, Facultad de Ciencias de Ourense, Universidad de Vigo, 32004 Ourense, Spain; 11 Department of Biotechnology and Sustainability, Technical University of Munich, Campus Straubing for Biotechnology and Sustainability, Essigberg 3, 94315 Straubing, Germany; shahidasiddiqui777@gmail.com; 12 DIL eV—German Institute of Food Technologies, Prof.-von-Klitzing-Straße 7, 49610 D-Quakenbrück, Germany; 13 National Institute of Food Science and Technology, University of Agriculture, Faisalabad 38000, Pakistan; raheemuaf@gmail.com

**Keywords:** pectin, average side chain length, rheology, LF-NMR, cookies

## Abstract

Pectin has been widely used as a hydrocolloid in foods, but its effectiveness based on hydrodynamics radius (Rh), average side chain length (ACL) and degree of esterification (DE) has been less studied. This study investigated the effect of 4 types of pectin (with different molecular weight and structures) at a level of 1.5% *w/w* of wheat flour on functional, structural and water binding properties of sugar snap cookies partially substituted with fish meat. The results showed that pectin (CU-201 and CU-601) with higher ACL and Rh controlled excessive expansion, while the improved rheology of dough in terms of behavior as viscous matrix compared to control and other pectin. Texture was found to be highly dependent on Rh and ACL compared to DE of pectin. The pasting properties, especially peak viscosity and final viscosity, were significantly (*p* < 0.05) increased with increasing DE, as well as ACL, by entangling and increasing the interaction between starch and pectin. The scanning electron microscopy (SEM) analysis exhibited that control sample showed wide voids and more intercellular spaces, while samples prepared with CU-601, CU-201, and CUL displayed compact structure, which was also evidenced by controlled expansion and improved hardness of the cookies. Low field nuclear magnetic resonance (LF-NMR) analysis showed that T21 relaxation time and amplitude were found to be shorter for CU-601 and CU-201 treatments, signifying the high amount of tightly bound water compared to control. The findings endorse the feasibility of adding CU-601, and CU-201 as an efficient hydrocolloid for the improved structural and functional properties of cookies.

## 1. Introduction

Snack foods have attracted a lot of attention and appeal because of their huge diversity in terms of ready-to-eat convenience, distinctive taste, price competitiveness, high nutrition, and long shelf life [1]. The starch–protein network, processing methods, and micronutrients present in the formulation all have a significant impact on the qualitative attributes of snack foods. These days, snack foods including cookies are made from various ingredients, e.g., fish meat [2], cricket powder [3], lupin seed protein [4], dietary fiber [5] for taste, and nutrition and as alternatives of traditional ingredients (wheat and rice flour). However, these novel ingredients influence mechanical stability, rheology, and physico-chemical as well as the structural properties of cookies. Thus, understanding of dough function and the evolution of the starch–protein network are critical considerations for optimizing cookies manufacturing.

In order to improve the mechanical stability and functional characteristics of finished goods, functional hydrocolloids and food additives, such as enzymes [6], antioxidants, emulsifiers [7], modified starches [8], gums, and more recently, pectin [9], are commonly used. These functional ingredients have been widely employed in a range of bakery and meat products, either alone or in mixture, to improve the quality, especially the textural, rheological and pasting qualities associated to sensory acceptability. Among these, hydrocolloids can be preferentially used in heat-induced processes to enhance the physicochemical characteristics (water binding throughout the product and water holding capacity) and change the interaction of hydrophilic and hydrophobic phases [10]. The synergistic nature could be due to the interaction of different hydrocolloids molecules with opposite or similar charges within food matrix. Oppositely charged hydrocolloids are likely to interact and produce a precipitate, but there is evidence that interaction results in gel formation for some stiff polysaccharide molecules [10]. Thus, by incorporating appropriate hydrocolloids into the food matrix, the qualitative attributes of goods can be restored.

Pectin, a polysaccharide found in plant cell walls, is widely used in the food, pharmaceutical, and biotechnology industries [11]. The versatile application of pectin stems from its complex molecular structure, diverse chain length distribution (mostly monosaccharides), degree of methyl esterification (DE), and hydrodynamic radius (Rh). Precisely, the diverse use of pectin arises from its complicated molecular structure, which consists mostly of galacturonic acid (Gal*p*A), rhamnose (Rha*p*), galactose (Gal*p*), arabinose (*Arap*), and other monosaccharide units. The monosaccharide composition consists of three main structural segments of pectic polysaccharides: homogalacturonan (HG, a linear polymer formed by 1, 4-linked α-D-galactopyranosyluronic acid residues), rhamnogalacturonan-I (*RG-I*, with repeating Gal*p*A-Rha*p* disaccharide blocks as backbones and side chains attached to Rha*p*), rhamnogalacturon (XGA) [8]. Primary molecular architectures (various monosaccharide compositions), molecular sizes, and DE of carboxyl groups in Gal*p*A can all change amongst pectin [11]. The physicochemical characteristics and physiological activities of pectin are intimately linked to its molecular structure. The physico-chemical and functional properties of pectin greatly depend on molecular weight and average side chain length distribution [9]. For example, pectin classic 051/13 (soluble apple pectin) and citrus peel with different DE (37 and 63%, respectively) and hydrodynamic radius (7.6 and 13 nm) have different gelling properties [9]. Thus, studying various types of pectin with different structural and functional characteristics is of high importance.

Numerous studies reveal the addition of pectin as hydrocolloids in snacks and starches [8,12], but the effect of various pectin with different structure and DE on structural and physiochemical properties of cookies has not been reported. In addition, the partial addition of dried fish meat powder in cookies is also novelty of our study. Since fish meat forms weak gel upon heating, thus, addition of pectin could strengthen the dough and starch-protein network. Therefore, the prime objective of this study was to investigate the effects of various pectin on dough rheology, microstructure, pasting and water binding properties of cookies that were prepared with partial addition of freeze-dried mince fish meat. The effectiveness of pectin was evaluated based on their average chain length, DE, and hydrodynamic radius. Finally, water binding within cookies was studied, and the effects with pectin and without pectin were explored. The study will provide meaningful information about the appropriate type of pectin to be used for cookies with diverse ingredients including meat. 

## 2. Materials and Methods

Four commercial pectin were used in this study: citrus peel pectin (CP, P9135, Lot# SSBS8828) was procured from Sigma-Aldrich Inc. (Natick, MA, USA). Pectin classic (CU-201 and CU-601) and pectin classic CU-L 051/13 (CUL) were gifted by Prof. Hans-Ulrich Endress Herbstreith and Fox KG, Germany, respectively. All-purpose high gluten wheat flour (moisture 10%, ash 0.45%, 12% protein content) of Jinsha River Industry Ltd., Chengdu, Sichuan, China, sucrose sugar, skimmed milk powder (brand: Devondale, Saputo Dairy Australia Pvt. Ltd., Australia Saputo, Allansford, Australia), salt and shortening were purchased from the local supermarket of Yangzhou University, Yangzhou, Jiangsu, China. The characteristics of pectin are shown in Table 1. Minced fish meat of Grass carp (*Ctenopharyngodon Idella*) was purchased from local supermarket and freeze-dried by means of freeze dryer using the following conditions: −70 °C, chamber pressure of 0.02 mbar for 24 h. Other chemicals used in this study were of analytical grade and used without further purification. 

### 2.1. Preparation of Cookies

Cookies were prepared according to the procedure of AACC (method No.10-52) and methodology of Zhang, et al. [12] with a small modification. Briefly, for control sample, 48 g sugar, 2.4 g skimmed milk powder, 0.8 g NaHCO_3_ were mixed together in order to make a cream by adding 24 g Cirsco^®^ vegetable shortening through micro-mixer (Bear Kitchen Aid, SJJ-B10T2, Jinhua, Zhejiang, China, 1 min for each at low (270 rpm), medium (360) and high speed (430 rpm), followed by the cleaning of the scrap blade after every step. Then, 8 mL solution A (79.8 g NaHCO_3_ in water to make final volume of 1 L) and 4 mL of solution B (101.6 g NH_4_Cl and 88.8 g NaCl in distilled water to make a final volume of 1 L) were added to 75.2 g of creamed mass with 17.4 mL of total water. This creamed mass was mixed for 3.5 min at medium speed, meanwhile 80 g wheat flour (moisture content: 14 g/100 g), 1 g freeze-dried fish meat was added, which was further mixed for 30 s. The dough was scraped from blades and bowl, cut into small portion and placed on pre-greased baking sheet. The sheet was cut into thickness of 15 mm and diameter of 55 mm using a stainless-steel shaper. The cookie dough was placed in a pre-greased baking paper, which was baked at 206 °C for 12 min in a baking oven (Model: HGB-20D, Rudong Jiahua Food machinery, Co., Ltd., Jiangsu, China). The baked cookies were subjected to cooling for 30 min at room temperature, which were stored in sealed plastic bags for further analysis. Similarly, for other treatments, pectin (1.5% *w/w* of wheat flour) was added along with wheat flour and a total of 5 treatments were prepared as follows: Control, CP, CUL, CU-201 and CU-601. 

### 2.2. Dynamic Rheological Properties of Dough

Dynamic rheological characteristics of dough or viscoelasticity of dough used for making cookies were assessed by a Discovery Hybrid Rheometer (HR1, TA instruments, Wakefield, MA, USA), which was attached with parallel plate geometry with a diameter of 40 mm following the methodology elsewhere [12]. Dough, as prepared for cookies, was loaded between parallel plates, and the gap was reduced to 1.5 mm. Paraffin oil was used to cover the edges in order to prevent moisture loss. The measurements were performed after 5 mints of loading in order to relax normal stresses produced during loading. A strain sweep (0.01–100%) at a fixed frequency of 1 Hz was used to measure the linear viscoelastic region of cookie dough. Rheological properties including storage modulus (G′), loss modulus (G′′) and tan δ were measured at 25 °C using a frequency range of 0.1–10 Hz at 1.5% strain within the linear viscoelastic region. Measurements were done in triplicate for accuracy, while graphs were plotted by calculating a mean value of triplicates. 

### 2.3. Diameter and Expansion 

Diameter and thickness (expansion) were measured before and after backing using a digital Vernier calliper (Mitutoyo Digital Caliper, Osaka, Japan), following the methodology of Nawaz, et al. [13]. Results were reported in the form of mean and standard deviation. For each treatment, six readings were recorded while mean values were reported. 

### 2.4. Texture Profile Analysis

A texture profile analysis of cookies was measured by TA-XT2 plus texture analyzer (TMS-TOUCH; Food Tech., Co., Slinfold, West Sussex, UK), which came from Stable Micro System, London, UK. The methodology of Zhang, Fan, Yang, Li, Gilbert and Li [12] was adopted using a three-point bending HDP/3PB probe with the following conditions: 20 mm probe travel distance, trigger force: 5 g, distance between two points of probe: 45 mm, penetrating distance: 5 mm, test speed 1.0 mms^−1^. Hardness (N) and fracturablity (mm) were calculated at the point of break in six cookies for each treatment. 

### 2.5. Pasting Properties 

Pasting properties of baked cookies (ground to make a powder using pulverizing mill (Hc-280T2, Yongkang Green Food Machiery Co., Ltd., Yongkang, Zhejiang, China) for 2 min to pass through a 60-mesh) were measured by a rapid visco analyzer (RVA) following the methodology of Nawaz, Xiong, Xiong, Irshad, Chen, Wang, Ahsan, Walayat, and Qamar [7]. All measurements were recorded in triplicate for each treatment, and mean values were reported. 

### 2.6. Scanning Electron Microscopy (SEM)

Scanning electron microscopy analysis of baked cookies was done using SEM (S-4800-II, Hitachi, Tokyo, Japan), as described in the study of Gu et al. [14]. All samples in the form of cross-section pieces were freeze-dried prior to analysis. A cross-sectional piece of cookies was placed on aluminum slid, which was fix by double-sticky edges and coated by gold before observation. All images were captured at a voltage of 10.0 KV and room temperature. 

### 2.7. Low Field Nuclear Magnetic Resonance (LF-NMR) Analysis of Cookies

LF-NMR analysis was done to evaluate the water binding of cookies inside the matrix, following the methodology of Bosmans et al. [15] with slight modifications. Briefly, a Minspec mq 20 Low-field pulsed NMR spectrometer of Bruker, Ettlingen, Germany was used to measure transverse relaxation time (T2) at 25 °C and 20 MHz, using a sequence based on Carr–Purcell-Meiboom–Gill (CPMG). The amount of proton in the population was proportional to the peaks obtained. Three regions: T21 (0.1–1 ms), T22 (1–100 ms), and T23 (100–1000 ms) were obtained and expressed in graphs.

### 2.8. Statistical Analysis

All experiments and analyses were performed in triplicate. The obtained data were processed for statistical analysis using SPSS (Version 19.0 for Windows (SPSS Inc., Chicago, IL, USA) in order to examine the statistically significant difference. All data were evaluated by one way analysis of variance (ANOVA) and Duncan multiple range’s test, at a significant level of *p* < 0.05.

## 3. Results and Discussion

### 3.1. Dynamic Rheological Properties of Dough

**Figure 1:**
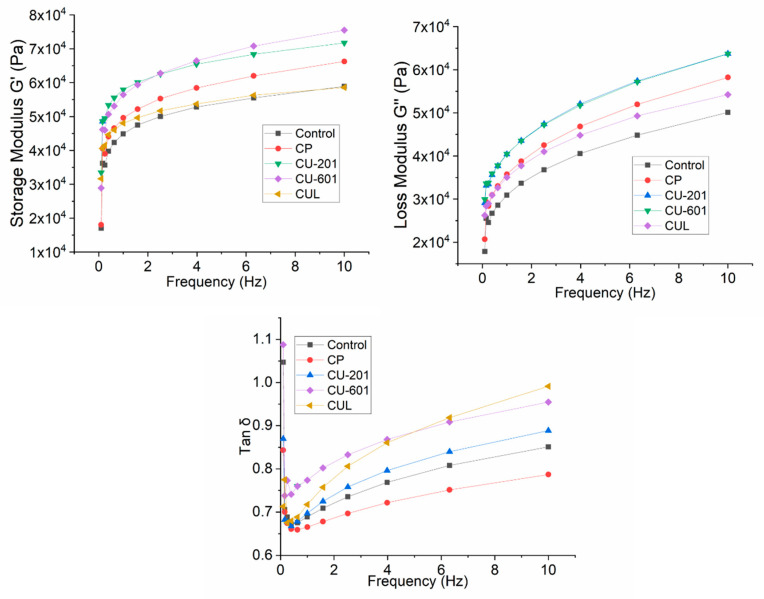
Rheological properties of cookies dough prepared with pectin having different molecular structure and molecular weight. CUL, CP, CU-601, and CU-201 indicate the names of pectin which were added in cookies. Reported graphs are the mean of triplicates for each treatment.

The properties and functional characteristics of pectin are highly dependent on source, bonding nature, monosaccharides units, functional groups, and extraction methods [9,16]. The results of dynamic rheological properties are shown in Figure 1. Storage modulus (G′), and loss modulus (G′′) were calculated in order to assess elastic solid or viscous liquid behavior. G′ and G′′ were increased with the increase in frequency. However, it was observed that G′ was increased in all samples, and it was highest in CU-601 followed by CU-201, while it was lowest in control followed by CUL. The same trend was observed for G′′, which was highest in CU-201 and CU-601 treatments, while it was lowest in the control sample. In addition to this, G′ > G′′ in all observed samples, suggesting the elastic solid-like behavior of cookie dough. This indicates that viscosity was increased with the addition of pectin as compared to the control sample, which ultimately indicated the strong interaction of the starch–protein network with pectin. With loss modulus (G′′), indicating the viscous properties, an increasing trend was observed for pectin samples, which was high in CU-201 and CU-601 treatments. G′′ was lower in the control sample followed by CUL batch, while it was highest in CU-201 and CU-601 groups, suggesting the low and high solution viscosity of two different pectins. Reflecting from the results of rheological properties, it can be suggested that CUL and CP treatments pectin might have formed entangled network instead of crosslinking, as dough behaved as a viscous matrix. In addition, the increase in ACL also caused more viscosity, which was also evidenced in CU-201 and CU-601 treatments, reaching maximum ACL compared to CP and CUL batches. However, some carboxylic groups, including methylesterified or uronic acid salt located in the HG and RG regions, influence the polymer charge balance in a solution [17]. Similarly, high DE inclines to form gel at lower pH, which is actually stabilized by hydrophobic interaction; on the other hand, low DE forms gel is electrostatically stabilized by metal cation [10]. The results of tan δ are also presented in Figure 1, which show that tan δ increased with the increase in frequency in the following order: CUL > CU601-CU201 > control > CP. Generally, lower value (close to 1) implies a more elastic solid-like behavior, while higher values indicate a rather viscous-like behavior, like solid behavior, while lower values indicated the thinning behavior. Liu, et al. [18] also reported the solid-like behavior of dietary fiber when added in dough. The increase in solid-like behavior with the addition of pectin can be explained in such a way that, during thermal processing, dietary fibers compete with starch and gluten for water, which reduce lubrication [19] and enhanced viscosity, as dietary fiber also acts as filler in the matrix [20]. Regarding rheological properties, the relative quantities of interconnected HG and RG-1 regions regulate extensibility and rheological properties: HG region induces the molecular interaction while RG-1 regions or branched regions indorse the development of entangled structures. Thus, it can be concluded that pectin (CU-201 and CU-601), with a higher number of side-chain length and higher molecular weight, interacted strongly with starch and protein matrix, which resulted in the improved rheological properties of dough.

### 3.2. Expansion 

**Figure 2:**
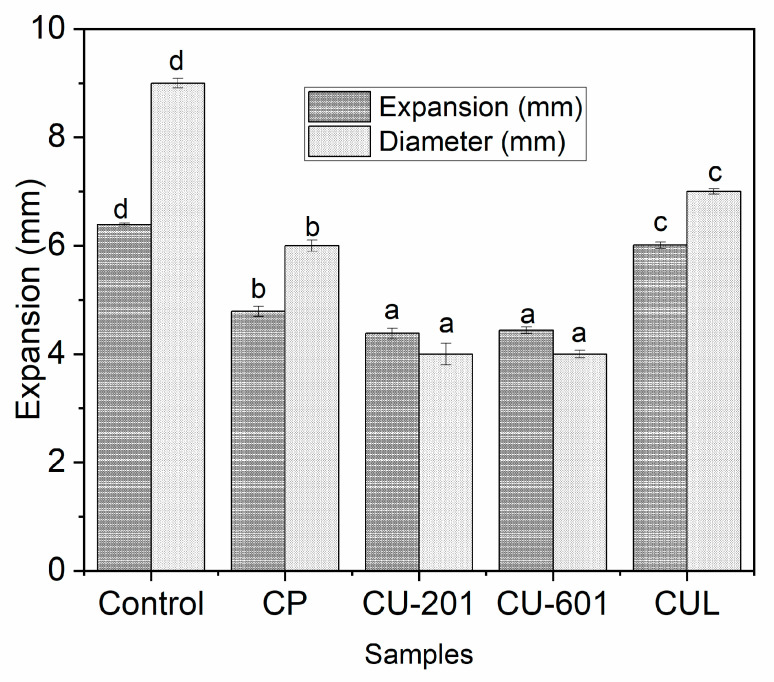
Expansion and changes in diameters of cookies dough prepared with pectin with different molecular structure and molecular weight. CUL, CP, CU-601, and CU-201 indicate the names of pectin which were added in cookies. Reported graphs are the mean of triplicates for each treatment. Small letters over error bars represent the significant difference between treatments at a level of *p* < 0.05 using one-way ANOVA and Duncan multiple range test.

In the present study, sugar snap cookies were prepared with the addition of 1% freeze-dried fish meat. Since cookies contain a low amount of water, thus, fresh meat was not added to the cookies. The addition of fish meat was done in the form of freeze-dried powder, in order to preserve the native characteristics of fish meat. Based on initial trails (date not shown here), a high amount of fish meat was not desirable, as fish meat proteins associate into weak gel upon heating. The results of expansion in terms of change in diameter and width (expansion) are displayed in Figure 2. Our findings revealed that expansion was significantly (*p* < 0.05) highest in the control group, followed by CUL and CP samples, while it was lowest in CU-201 and CU-601 treatments. There was no statistically significant (*p* < 0.05) difference between CU-201 and CU-601 groups. On the other hand, the same trend was observed for change in diameter, which was highest in control and lowest in CU-201 batch. The reason for the highest expansion in control might be due to the addition of fish meat in matrix, which interrupts the continuity of the gluten network and results in a weaker structure upon heating [21]. It is worth mentioning that only 1% of freeze-dried fish meat was added in each formulation, which is equal to 5% of fresh meat. A higher amount of fish meat was not possible, especially in cookies, as its shape and structure were disrupted, owing to the viscoelastic properties of fish meat upon heating. Even with the addition of 1% dried fish meat, there was extensive expansion, and thus, we added pectin to investigate the impact of various pectin for a smooth structure and physico-chemical properties. 

The addition of pectin improved the expansion and diameters, which controls not only the viscoelasticity of fish meat, but also controls the excessive unfolding of gluten protein upon heating [22]. The expansion values in CUL near to control samples might be associated with the anionic property of both hydrocolloids; consequently, repulsive forces can take place. However, this can be improved by adding some calcium that can improve the share stress of gel [10]. However, different pectins behave differently, which signifies the role of structural differences. Since excessive expansion in cookies is not desirable, the addition of pectin especially with higher chain length and molecular weight has potential to control excessive expansion of cookies fortified with fish meat. 

### 3.3. Texture Profile Analysis

**Figure 3:**
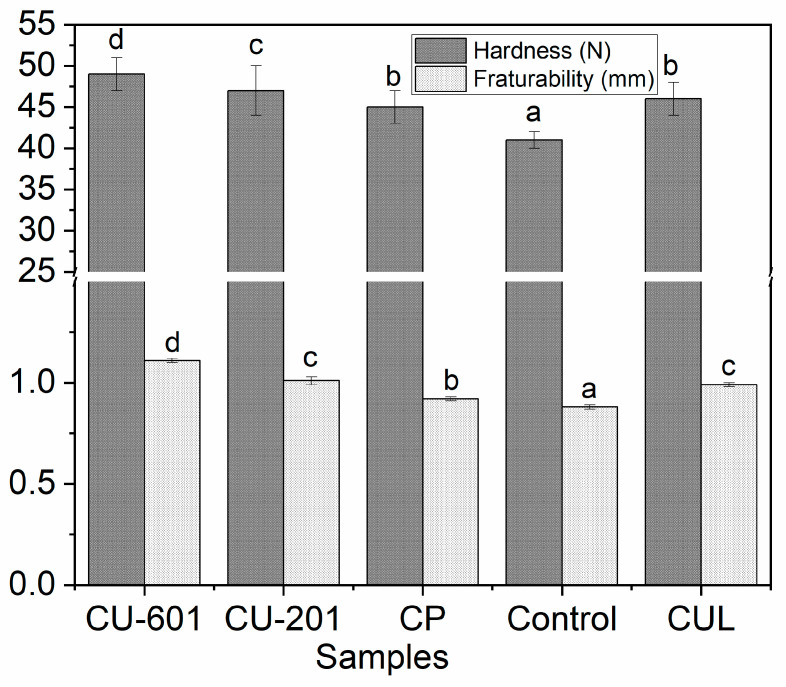
Textural analysis of cookies prepared with pectin with different molecular structure and molecular weight. CUL, CP, CU-601 and CU-201 indicate the names of pectin which were added in cookies. Reported graphs are the mean of triplicates for each treatment. Small letters over error bars represent the significant difference between treatments at a level of *p* < 0.05 using one-way ANOVA and Duncan multiple range test.

The texture of cookies is usually assessed by its hardness and fracturability. Hardness is defined as a substantial resistance to deformation when force is applied. Usually, hard texture is preferred in cookies, which is mainly because of sugar and less water in the formulation. The results of texture profile analysis are given in Figure 3. It was disclosed that control sample showed significantly (*p* < 0.05) less hardness, indicating the fragile or loose structure. Even this hardness was less than the hardness of snacks prepared from 5% whole fish meat [23]. The decreased hardness was associated with the addition of fish meat and gluten protein that show viscoelastic properties upon heating. However, this hardness was improved when pectin was added into formulation. Significant increase (*p* < 0.05) in hardness was observed in pectin sample, and it was maximum in CU-601 treatment followed by CU-201 batch. On the other hand, there was no statistically significant (*p* < 0.05) difference between samples, which were prepared by the addition of CP and CUL. DE of pectin plays a great role in the hardness of the end-product. Some studies reported that flexibility is directly proportional to DE, while rigidity is inversely proportional to the DE of pectin [17]. However, our results are in contrast with previous study [17]: a higher degree of DE resulted in increased hardness. This difference might be due to the different use and processing method for which pectin has been added. The reason on contrast to this study might be due to another phenomenon: hardness is inversely proportional to expansion [23]. The increased hardness as a result of less interstitial spaces and compact structure results in the decreased expansion. From these findings and property of pectin (dietary fiber) to be used as filler in thermal processing, it can be inferred that pectin might improve the hardness of cookies by decreasing the intercellular space within the matrix, which was also evidenced by a previous study that dietary fiber inclusion increased the hardness [24,25]. The improved hardness may be due to hydrogen bonds between pectin with DE and myofibrillar protein, as reported elsewhere [10]. In addition, due to high molecular size and chain length, the branched structure of CU-201 and CU-601 groups promoted the cross-linking reaction, which resulted in increased hardness. 

### 3.4. Pasting Properties

Pasting properties predict the gelling ability of material and ability to withstand during thermal processing. In addition, gelatinization characteristics of starch in heterogeneous system determine the stability and structural attributes of end product. Moreover, the extent of starch–protein interaction during thermal processing can better predict the internal interaction of ingredients within the heterogeneous system. Usually, the pasting behavior of dough is determined, but our aim was to assess the stability of pectin after heating. 

**Figure 4:**
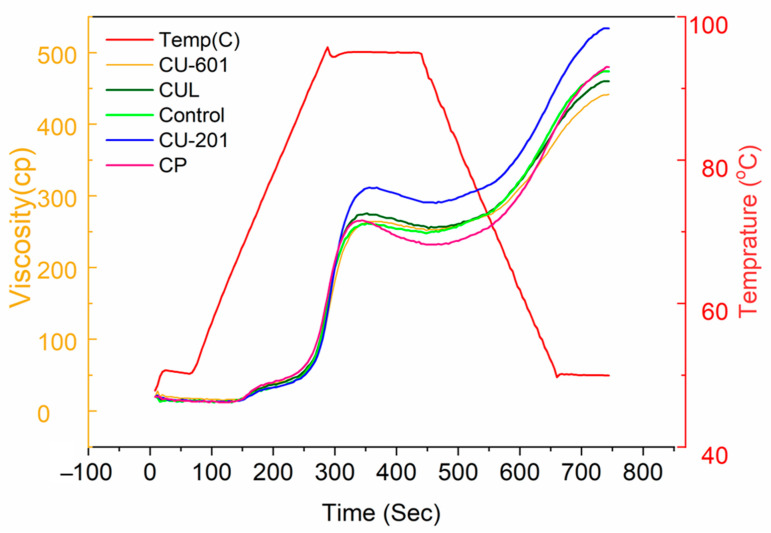
Pasting properties of cookies prepared with pectin, with different molecular structures and molecular weights. CUL, CP, CU-601, and CU-201 indicate the names of pectin which were added in cookies.

The pasting properties of cookies prepared with and without pectin and partially added fish meat are shown in Figure 4 and Table 2. The results showed that peak viscosity was significantly (*p* < 0.05) increased in those samples prepared with pectin (CU-201 and CU-601 treatments), presenting higher average side chain length and hydrodynamic radius (Rh). On the other hand, it was noticed that control sample displayed lowest peak viscosity followed by CP and CUL groups. It was obvious that the addition of pectin increased the peak viscosity as compared to control in line with a previous study [16], but our aim was to screen the best gelling ability of the studied pectin based on their structural differences for snack food. Since peak viscosity reflects the binding strength of ingredients at the molecular level, it is possible that longer side chain (as seen for CU-201 and CU-601 treatments) promoted the interaction within matrix and peak viscosity increased. Moreover, peak viscosity is related to the paste formation, mostly by starch due to its gelatinization and the addition of hydrocolloids, such as pectin, can contribute to the increase of this parameter, due to the increasing viscosity of the formed paste. In this regard, Luo, Chen, Huang, Liang, Liu, and Chen [16] proposed that structural features greatly influence the pasting properties of pectin: 10% increase in degree of methoxylation (DM) of pectin resulted in improved peak viscosity of rice starch, but degree of amidation (DA) negatively affected the peak viscosity and breakdown values. This strongly suggests the dependence of physiochemical and structural characteristics on molecular size, weight as well as the degree of esterification and amidation [26]. The reason for the increased pasting viscosities in case of more DM could be as follows: as DM of pectin increases, ability of pectin to compete for water decreases, which would result in a greater availability of water for starch, suggesting an increase in pasting properties. 

Trough viscosity indicates the stability of paste during the cooling process. The results revealed a different trend compared to peak viscosity and was in the following order: CU-601 > CU-201 > control > CUL > CP. Breakdown viscosities (generally, lower values are believed to be good indication) were found to lower in CU-601 and CU-201 treatments, but no statistically significant (*p* < 0.05) difference was noticed between these two samples. Only one significant difference was observed between control and CP batches. It was interesting to note that the CP group showed highest breakdown values even greater than control samples. Final viscosities and setback vales indicated the strength of starch granules to withstand and degree of rearrangements of starch granules after gelatinization, respectively [23]. The observation of final viscosity and setback indicated that there was statically significant difference (*p* < 0.05) in samples. Minimum final viscosity was found in control sample compared to all samples, indicating the additive effect of pectin, which promoted crosslinking of starch within matrix. However, there was no statically significant difference between CUL and CU-601 treatments. The setback values highly depend on molecular weight and are positively related to molecular weight [27]. Generally, higher molecular weight and long side chain entangle during thermal processing, wriggle and disentangle more quickly, which make a viscous matrix and provide resistance to flow. Our results agree with the previous studies conducted on the effects of molecular weight of dietary fibers (pectin and xanthan gum) on the pasting properties of starch [27,28,29]. This might be due to the fact that higher molecular weight of pectin will have stronger thermodynamic compatibility among starch granules and pectin [28]. Thus, having higher molecular weight and especially the number of side chain could effectively increase the setback of cookies. 

The findings of peak time and temperature disclosed that peak time for CU-601 treatment was minimum compared to all samples, while it was highest in the control group. However, CP and CUL batches showed no statistically significant difference (*p* < 0.05) when compared with other samples. Similarly, for peak temperature, which is an indication of cooked food, was found to be the lowest in CU-601, whereas it was highest in control. The reason for the decreased time and temperature for the above pectin sample might be that these pectins acted as the best fillers in the matrix, which reduced the intercellular spaces between molecules by crosslinking or interacting with other ingredients of formulation; thus, more heat was transferred through solids, which reduced the temperature and time. Summarizing the findings of pasting properties, it can be inferred that DE and ACL are important factors that determine the pasting properties of pectin, and samples (CU-601 and CU-201 batches) with longer ACL and DE showed the best possible pasting properties.

### 3.5. Microstructure

**Figure 5:**
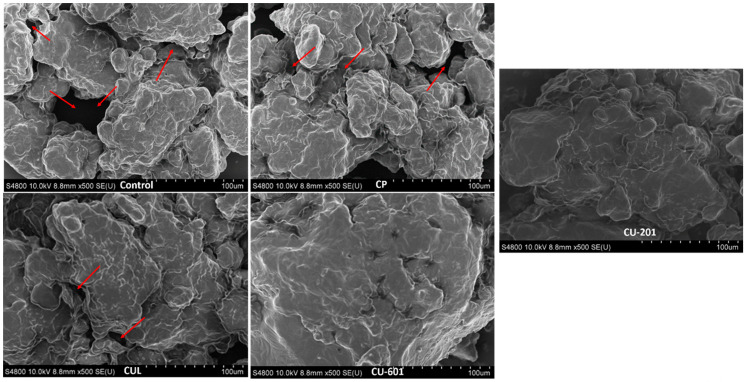
Microstructure of cookies prepared with pectin, with different molecular structures and molecular weights. CUL, CP, CU-601, and CU-201 indicate the names of pectin which were added in cookies. Red arrows show the interstitial spaces within matrix.

Microstructure represents and verifies the other findings at molecular level. The results for microstructure of cookies for all samples are shown in Figure 5. Micrographs were taken at a same magnification level for uniformity and consistency. Control sample showed more voids and scattered structure indicating more interstitial spaces within the matrix. These interstitial spaces were also evidenced in terms of increased expansion of control samples and less hardness, as high expansion (bubble formation) caused more intercellular spaces. Some irregular particles were uniformly distributed in the control sample. This phenomenon can be explained as during thermal processing, starch molecules swell to their maximum size prior to fragmentation owing to the hydration of water. The maximum expansion in control sample might be attributed to the lack of pectin, which creates competition with starch granules for water molecules; thus, the absence of pectin creating excessive expansion. However, excessive expansion is not desirable in cookies, which can deform the shape. Another possibility for higher expansion in control samples might be fish meat, which could not crosslink with starch and gluten protein, increased air cell size, and decreased the bulk density, which was also observed in terms of low hardness in control sample. The addition of pectin controlled this excessive expansion, which was also observed in terms of less voids and holes, ultimately improving hardness and bulk density. Bulk density was not measured, but from hardness and expansion, it can be postulated that bulk density would be improved, as samples with pectin expanded less compared to control.

The compact structure was observed in CU-601 and CU-201 with embedded starch and protein molecules. This compact structure might be due to the crosslinking and interaction between pectin and starch–protein matrix by means of esterified homogalacturonic acids regions and hydrophobic bonding [10]. This was also evidenced in expansion values, which were decreased for CU-601 and CU-201 for control. 

### 3.6. Low Field Nuclear Magnetic Analysis (LF-NMR) Analysis

The water binding and water mobility properties are monitored by measuring the relaxation time values (T_2_) by means of LF-NMR analysis, which can be divided into three regions: T_21_ ranging from 0.1–1 ms indicates tightly bound water. Meanwhile, T_22_ (1–100 ms) corresponds to immobilized water and T_23_ (100–1000 ms) signifies the unbound water. It is believed that short relaxation time and amplitude indicate the strong association between solids and water within matrix. 

**Figure 6:**
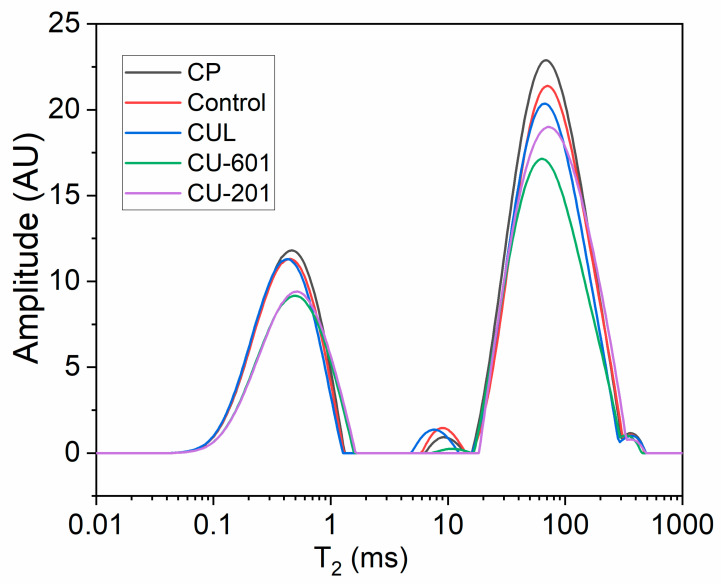
LF-NMR analysis of cookies prepared with partially added fish meat and various types of pectin. CUL, CP, CU-601, and CU-201 indicate the names of pectin which were added to the cookies.

The results of LF-NMR relaxometry analysis of cookies prepared with diverse types of pectin are given in Figure 6. All samples showed 3 major peaks showing different compartments of water. This number of water regions is consistent with previous studies [7,23]. According to results, T_21_ amplitude and relaxation time were highest in CP followed by control, suggesting that less tightly bound water was found for these samples; whereas, CU-601 and CU-201 treatments displayed short relaxation time and amplitude, which implies that water was tightly bounded within matrix for these samples. The high proportion of tightly bound water in CU-601 and CU-201 samples may be due to the hydrophilic nature of these types of pectin. As discussed earlier, if molecules are closely arranged or have a shorter distance, a high energy efficiency is achieved, which will produce shorter T_2_ relaxation time [30]. Similar findings were observed by Han and Bertram [31] when pectin was added to meat-based products. The dominance of T_21_ relaxation time for the above said sample might be due to higher DE and DM, which created strong thickness of aqueous layers around starch and pectin molecules. On the other hand, high amplitude and relaxation time in CP and control samples may have some surface activity or slightly hydrophobic behavior.

Water binding in cookies is usually assessed by T_21_ and T_22_ proton population, owing to the limited amount of water in cookies [3]. However, in our study, cookies were prepared with the addition of fish meat, which could behave differently; thus, we divided water binding into 3 groups, considering the possibility of high amount of water retained inside the matrix after baking [32]. T_22_ relaxation region showed fewer peaks in all samples. However, from results, it was evidenced that only CU-601 treatment showed minimum relaxation amplitude and time, suggesting the presence of a high percentage of immobilized water in this sample. Regarding T_23_ region, two peaks were observed, however, due to short relaxation time and amplitude from 900–1000 ms, a smaller peak was ignored with no significant effect. This T_23_ region is very important, which displayed unbound water. The presence of a high amount of unbound water affected the textural, gelling and shelf-life characteristics. Moreover, unbound water causes rapid deterioration and increases the risk of microbial growth under favorable condition, as well as facilitating mold growth. A longer relaxation time and amplitude were observed for CP group followed by control, whereas shorter relaxation time and amplitude were observed for CU-601 treatment followed by CU-201 batch. This might be attributed to the strong crosslinking and entanglement of these pectin with starch and protein molecules, which promoted the gelling behavior and improved the rheological properties, as evidenced in rheology and pasting properties of these respective pectin. A previous study also showed that crosslinking promotes rigid behavior [33], which leads towards the formation of compact structure, increased hardness, decreased expansion, and improved pasting properties.

## 4. Conclusions

The study was done to get deep insights into the influence of molecular weight and average chain length, as well as degree of esterification of various pectin on the structural and physio-chemical properties of cookies prepared with partially substituted freeze-dried fish meat. The major findings revealed that molecular weight in terms of Rh and ACL were important considerations, which greatly affected the rheological properties of dough with the recommendation of higher Rh and ACL of pectin, as seen for this study. These findings can be used as a reference for other snack foods. Texture was found to be highly dependent on DE and improved in case of higher DE; however, it was conflicted from previous studies. Pasting properties were improved for those samples prepared with pectin having high ACL, which crosslinked and entangled, resulting in more viscous and less lubrication in the matrix.

The findings suggest the feasibility of adding CU-601 and CU-201 pectin as an efficient hydrocolloid for cookies for a proper structure, texture and water binding properties. There are some limitations of this study: the study did not include the investigation of DM of pectin which could be a future prospective of this study. In addition, the comparative study of pectin with other hydrocolloids with different Rh, ACL and DE for cookies (especially meat-based) can be another future prospective of this study. With all these implications, the addition of pectin can be described as a suitable hydrocolloid for improving the physiochemical and structural properties of cookies.

## Figures and Tables

**Table 1:** Structural characteristics of pectin samples.

Samples/Parameters	Degree of Esterification (%)	Average Side-Chain Length	Average R_h_ (nm)
CP	63.8 ± 2.7 ^c^	2.8 ± 0.1 ^a^	13.7 ± 1.6 ^a^
CUL	36.6 ± 3.5 ^a^	3.1 ± 0.2 ^a^	14.4 ± 1.8 ^ab^
CU-201	68.2 ± 1.4 ^d^	4.9 ± 0.3 ^c^	16.9 ± 1.9 ^c^
CU-601	53.7 ± 2.7 ^b^	3.7 ± 0.7 ^b^	15.2± 1.7 ^b^

Small letters (a–d) represent the significant difference within rows using one way ANOVA and Duncan’s Multiple range test. Reported results are mean and standard deviation of triplicates.

**Table 2:** Pasting properties of cookies prepared with pectin with different molecular structure and molecular weight.

Test/Samples	CU-601	CU-201	Control	CP	CUL
Peak viscosity (RVU)	294 ± 10.32 ^c^	312 ± 11.45 ^d^	276 ± 09.61 ^b^	266 ± 5.28 ^a^	292 ± 15.50 ^c^
Trough Viscosity (RVU)	256 ± 6.97 ^ab^	290 ± 11.93 ^c^	251 ± 11.89 ^ab^	232 ±15.74 ^a^	248 ± 13.20 ^b^
Breakdown Viscosity (RVU)	12 ± 6.38 ^a^	12 ± 8.99 ^a^	21 ± 3.50 ^c^	34 ± 8.60 ^d^	14 ± 5.33 ^ab^
Final Viscosity (RVU)	471 ± 13.44 ^c^	534 ± 30.50 ^d^	430 ± 15.44 ^a^	450 ± 10.22 ^b^	474 ± 17.34 ^c^
Setback (RVU)	207 ± 12.50 ^c^	222 ± 15.87 ^d^	184 ± 06.44 ^a^	194 ± 06.28 ^b^	202 ± 11.87 ^bc^
Peak Time (min)	5.73 ± 0.20 ^a^	5.93 ± 0.10 ^c^	6.00 ± 0.11 ^d^	5.88± 0.09 ^b^	5.83± 0.20 ^b^
Pasting Temperature (°C)	92.23± 0.22 ^ab^	92.12 ± 0.09 ^ab^	93.76± 0.11 ^d^	93.23 ± 0.20 ^c^	92.98 ± 0.10 ^b^

The results of pasting properties are mean and standard deviation of triplicates of each treatment, respectively. Different letters (a–d) in rows indicate the significant difference between processing methods at *p* < 0.05 using one way ANOVA and Duncan’s multiple range test CUL, CP, CU-601, and CU-201 indicate the names of pectin, which were added in cookies.
